# Supplementary material for: COVID-19 symptom load as a risk factor for chronic pain: A national cross-sectional study
Source: PLoS One. 2023 Jun 23;18(6):e0287554. doi: 10.1371/journal.pone.0287554 (PMC10289324; doi:10.1371/journal.pone.0287554)
Supplement: S1 Table — Covariates distributions were compared using Chi-Square tests, and most were found to be imbalanced amongst the COVID-19 groups prior to matching. After matching, no significant differences were found, and all standardized mean differences were <0.1, indicating good balance. (DOCX) [file pone.0287554.s001.docx]

**S1 Table: Covariate distribution amongst COVID-19 groups before and after matching**

| **Characteristics** | **Weighted, Unmatched** | | | | **Weighted, Matched** | | | | **Standardized Mean Differences** | | |
| --- | --- | --- | --- | --- | --- | --- | --- | --- | --- | --- | --- |
|  | **COVID -** | **COVID+, A/Mi** | **COVID+, Mo/S** | **Chi-Square p-value** | **COVID-** | **COVID+, A/Mi** | **COVID+, Mo/S** | **Chi-Square p-value** | **COVID- vs. COVID+ A/Mi** | **COVID+ A/Mi vs. COVID+ Mo/S** | **COVID+ Mo/S**  **vs. COVID-** |
| **Age Decades** | |  |  |  |  |  |  |  |  |  |  |
| 18-29 | 21.85% | 28.51% | 21.66% | <0.0001 | 27.51% | 27.51% | 26.05% | 0.94 | 0.00 | 0.03 | -0.03 |
| 30-39 | 17.65% | 20.12% | 18.99% |  | 19.92% | 20.73% | 19.58% |  | -0.02 | 0.02 | -0.01 |
| 40-49 | 16.00% | 17.91% | 20.41% |  | 18.53% | 18.50% | 21.11% |  | 0.00 | -0.05 | 0.05 |
| 50-59 | 15.89% | 14.56% | 18.10% |  | 15.21% | 14.88% | 14.53% |  | 0.01 | 0.01 | -0.02 |
| 60-69 | 15.27% | 8.95% | 12.14% |  | 10.08% | 8.88% | 10.30% |  | 0.03 | -0.04 | 0.01 |
| 70-79 | 9.52% | 7.09% | 6.49% |  | 6.71% | 6.96% | 6.35% |  | -0.01 | 0.02 | -0.01 |
| 80+ | 3.82% | 2.87% | 2.20% |  | 2.04% | 2.54% | 2.07% |  | -0.03 | 0.03 | 0.00 |
| **Sex** |  |  |  |  |  |  |  |  |  |  |  |
| Female | 53.02% | 52.65% | 55.87% | 0.16 | 52.14% | 52.32% | 53.44% | 0.84 | 0.00 | -0.02 | 0.02 |
| Male | 46.98% | 47.35% | 44.13% |  | 47.86% | 47.68% | 46.56% |  | 0.00 | 0.02 | -0.02 |
| **Race Ethnicity** | |  |  |  |  |  |  |  |  |  |  |
| Non-Hispanic White | 61.39% | 55.73% | 59.27% | <0.0001 | 60.09% | 57.74% | 59.27% | 0.96 | 0.04 | -0.03 | -0.01 |
| Non-Hispanic Black | 13.21% | 11.41% | 11.08% |  | 10.19% | 10.15% | 10.84% |  | 0.00 | -0.02 | 0.02 |
| Hispanic | 16.72% | 25.65% | 24.60% |  | 24.69% | 26.19% | 24.74% |  | -0.03 | 0.03 | 0.00 |
| Asian | 6.28% | 4.27% | 2.53% |  | 3.15% | 2.98% | 2.90% |  | 0.01 | 0.00 | -0.01 |
| Other single or multiple races | 2.38% | 2.94% | 2.51% |  | 1.88% | 2.94% | 2.25% |  | -0.06 | 0.03 | 0.02 |
| **Education** | |  |  |  |  |  |  |  |  |  |  |
| HS Graduate or less | 32.61% | 43.24% | 40.74% | <0.0001 | 41.24% | 42.31% | 40.58% | 0.89 | -0.02 | 0.03 | -0.01 |
| Some College, no degree | 14.83% | 16.39% | 18.08% |  | 16.65% | 15.91% | 16.88% |  | 0.02 | -0.02 | 0.01 |
| Associates (academic or vocational) | 11.39% | 11.10% | 11.67% |  | 10.59% | 11.65% | 12.51% |  | -0.03 | -0.02 | 0.05 |
| Bachelor's Degree | 25.23% | 19.55% | 20.48% |  | 22.56% | 20.28% | 20.87% |  | 0.05 | -0.01 | -0.03 |
| Higher Degree | 15.94% | 9.71% | 9.03% |  | 8.97% | 9.85% | 9.17% |  | -0.02 | 0.02 | 0.01 |
| **Poverty Income Ratio** | | |  |  |  |  |  |  |  |  |  |
| 0-1.24 (low) | 12.44% | 16.00% | 16.02% | <0.0001 | 14.96% | 14.36% | 15.91% | 0.84 |  |  |  |
| 1.25-2.99 (middle income) | 27.67% | 30.12% | 31.92% |  | 30.82% | 29.12% | 29.94% |  | 0.01 | -0.04 | 0.02 |
| 3.0-5 (high income) | 59.89% | 53.89% | 52.06% |  | 54.22% | 56.52% | 54.16% |  | 0.03 | -0.01 | -0.02 |
| **BMI** |  |  |  |  |  |  |  |  | -0.04 | 0.04 | 0.00 |
| Underweight | 1.79% | 2.26% | 0.78% | <0.0001 | 0.73% | 1.20% | 1.07% | 0.42 |  |  |  |
| Healthy weight | 32.34% | 30.78% | 23.85% |  | 29.25% | 30.53% | 27.17% |  | -0.04 | 0.01 | 0.03 |
| Over weight | 33.81% | 35.45% | 33.29% |  | 35.44% | 35.74% | 34.10% |  | -0.02 | 0.06 | -0.04 |
| Obese | 32.05% | 31.51% | 42.08% |  | 34.58% | 32.53% | 37.67% |  | -0.01 | 0.03 | -0.02 |
| **Arthritis Diagnosis** | 19.95% | 14.35% | 22.25% | <0.0001 | 14.40% | 14.71% | 16.29% | 0.44 | 0.04 | -0.09 | 0.05 |
| **Weakened Immune System** | 4.78% | 3.79% | 5.69% | 0.08 | 4.11% | 3.43% | 4.60% | 0.37 | -0.01 | -0.04 | 0.04 |
| **Diabetes** | 8.87% | 7.70% | 10.57% | 0.05 | 5.92% | 5.91% | 5.70% | 0.97 | 0.03 | -0.05 | 0.02 |
| **Difficulty Walking/ Functional Limitations** | | | | |  |  |  |  | 0.00 | 0.01 | -0.01 |
| No/Some | 95.95% | 98.09% | 94.70% | <0.0001 | 98.87% | 98.92% | 98.21% | 0.28 |  |  |  |
| A lot/Cannot walk | 4.05% | 1.91% | 5.30% |  | 1.13% | 1.08% | 1.79% |  | 0.00 | -0.05 | 0.04 |

HS = High School; BMI = Body Mass Index; A/Mi = Asymptomatic/Mild; Mo/S = Moderate/Severe
