# Supplementary material for: COVID-19 symptom load as a risk factor for chronic pain: A national cross-sectional study
Source: PLoS One. 2023 Jun 23;18(6):e0287554. doi: 10.1371/journal.pone.0287554 (PMC10289324; doi:10.1371/journal.pone.0287554)
Supplement: Attachment — Submitted filename: Recom.docx [file pone.0287554.s002.docx]

It is no doubt that the author included a huge data and this will increase the reliability of the results but I wonder why he ignored some an important risk factors such as smoking, drinking, and hypertension. Anyhow I think this manuscript is eligible for publishing as it.
