# Supplementary material for: COVID-19 symptom load as a risk factor for chronic pain: A national cross-sectional study
Source: PLoS One. 2023 Jun 23;18(6):e0287554. doi: 10.1371/journal.pone.0287554 (PMC10289324; doi:10.1371/journal.pone.0287554)
Supplement: Attachment — Submitted filename: Response to Reviewers.docx [file pone.0287554.s004.docx]

We thank the Editor and Reviewers for their comments and time. Below are our responses to their additional comments. In addition, we have reformatted our references.

**Additional Editor Comments:**

This is an excellent paper. However, the authors did not address my previous comments.
RESPONSE: We thank the editor for this comment. Please know that we did not see the three comments below included in the original request for revision.

#1. IRB review was exempt for this study. -> The University IRB is exempt, but there is a CDC IRB for the National Health Interview Survey. Describe it.
RESPONSE: We have added a line about the NCHS review board.

#2. Ref 18 and 19 is too old (1985??). Please cite this paper. DOI: https://doi.org/10.54724/lc.2022.e18
RESPONSE: Even though the publication dates on these two manuscripts are older, these are foundational manuscripts for propensity scores. We believe these maintain their importance, and have elected to keep these foundational references. Please note that in addition, we also cite newer yet renowned manuscripts that, in tandem, provide instructional support for readers. At the editor’s specific request, we have added the following citation:
Lee SW, Acharya KP. Propensity score matching for causal inference and reducing the confounding effects: statistical standard and guideline of Life Cycle Committee. Life Cycle. 2022;2:e18.

#3. This is an excellent paper!

RESPONSE: We thank the editor for their time.


**Reviewers' comments:**

Reviewer #1: In regard to my comments, the author's answers convinced me.
All required questions have been answered and that all responses meet formatting specifications.
RESPONSE: We thank the reviewer for their time.

Reviewer #2: - In table 1: you mentioned % in the head rows and not need the % after the figure for each individual cell.
RESPONSE: We have identified multiple manuscripts published within PLOS ONE that format tables with % signs included within the cells, and the column header as %. We have elected to maintain this original format.

Reviewer #3: All comments have been addressed. Clarifications have been added, and the methods and results look clearer.
Great paper. Well done to Authors!
RESPONSE: We thank the reviewer for their time.
